# *In vitro* development of resistance against antipseudomonal agents: comparison of novel β-lactam/β-lactamase inhibitor combinations and other β-lactam agents

**DOI:** 10.1128/aac.01363-23

**Published:** 2024-03-25

**Authors:** Mariana Castanheira, John H. Kimbrough, Jill Lindley, Timothy B. Doyle, Jessica M. Ewald, Helio S. Sader

**Affiliations:** 1 JMI Laboratories, North Liberty, Iowa, USA; 2 The Jackson Laboratory for Genomic Medicine, Farmington, Connecticut, USA; Universita degli studi di roma La Sapienza, Rome, Italy

**Keywords:** *P. aeruginosa*, β-lactams, mutations

## Abstract

We subjected seven *P*. *aeruginosa* isolates to a 10-day serial passaging against five antipseudomonal agents to evaluate resistance levels post-exposure and putative resistance mechanisms in terminal mutants were analyzed by whole-genome sequencing analysis. Meropenem (mean, 38-fold increase), cefepime (14.4-fold), and piperacillin-tazobactam (52.9-fold) terminal mutants displayed high minimum inhibitory concentration (MIC) values compared to those obtained after exposure to ceftolozane-tazobactam (11.4-fold) and ceftazidime-avibactam (5.7-fold). Fewer isolates developed elevated MIC values for other β-lactams and agents belonging to other classes when exposed to meropenem in comparison to other agents. Alterations in *nalC* and *nalD,* involved in the upregulation of the efflux pump system MexAB-OprM, were common and observed more frequently in isolates exposed to ceftazidime-avibactam and meropenem. These alterations, along with ones in *mexR* and *amrR,* provided resistance to most β-lactams and levofloxacin but not imipenem. The second most common gene altered was *mpl,* which is involved in the recycling of the cell wall peptidoglycan. These alterations were mainly noted in isolates exposed to ceftolozane-tazobactam and piperacillin-tazobactam but also in one cefepime-exposed isolate. Alterations in other genes known to be involved in β-lactam resistance (*ftsI*, *oprD*, *phoP, pepA,* and *cplA*) and multiple genes involved in lipopolysaccharide biosynthesis were also present. The data generated here suggest that there is a difference in the mechanisms selected for high-level resistance between newer β-lactam/β-lactamase inhibitor combinations and older agents. Nevertheless, the isolates exposed to all agents displayed elevated MIC values for other β-lactams (except imipenem) and quinolones tested mainly due to alterations in the MexAB-OprM regulators that extrude these agents.

## INTRODUCTION

*Pseudomonas aeruginosa* is an opportunistic pathogen and a leading cause of nosocomial infections. This organism is intrinsically resistant to multiple antimicrobial classes limiting the therapeutic options to treat pseudomonal infections. Although resistance to antimicrobial agents with antipseudomonal activity can be acquired *via* horizontal gene transfer, the accumulation of mutations reducing antimicrobial target binding or modulating the expression of constitutive genes are the primary avenues to resistance in *P. aeruginosa* (1). These mechanisms can be targeted to a single antimicrobial agent or, in the case of overexpression of efflux pumps or acquisition of genetic mobile elements containing multiple resistance genes, the susceptibility of multiple antimicrobial classes can be affected. Resistance to three or more antimicrobial classes—categorized as multidrug resistance (MDR)—is common among *P. aeruginosa* (2). MDR rates among clinical *P. aeruginosa* strains collected during a 20-year global surveillance study ranged from 23.7% to 44.6% depending on the type of infection and geographic region (3). The Centers for Disease Control and Prevention reports that MDR *P. aeruginosa* caused over 32,000 cases of infections in hospitalized patients in 2017 leading to the death of 2,700 patients (4).

The β-lactam/β-lactamase inhibitor combinations (β-LICs) ceftolozane-tazobactam and ceftazidime-avibactam have become mainstays in the treatment of MDR *P. aeruginosa* infections (5, 6). The β-lactamase inhibitor protects the cognate β-lactam from hydrolysis by the constitutively expressed chromosomal AmpC (7) and acquired β-lactamases. Despite the advantage against β-lactamase-mediated resistance, other common resistance mechanisms used by *P. aeruginosa*, including overexpression of efflux pumps, reduced membrane permeability, and target alteration, still result in β-lactam resistance with or without a β-lactamase inhibitor present. In addition, modified β-lactamases resistant to β-lactamase inhibitors have been reported (8–10).

Several studies describe antipseudomonal β-lactam resistance mechanisms, including among isolates resistant to these newer β-lactam/β-LICs (9–19); however, fewer studies have addressed the effect of exposing *P. aeruginosa* clinical isolates to these agents, specifically their effect on promoting the development of resistance compared to older agents. In this study, we subjected seven clinical *P. aeruginosa* isolates (Table 1) with low antipseudomonal β-lactam MIC values to a 10-day serial passage with one of the following five agents: ceftazidime-avibactam, ceftolozane-tazobactam, meropenem, cefepime, and piperacillin-tazobactam. We then used whole-genome sequencing (WGS) to identify putative resistance mechanisms to better understand the potential effects of prolonged exposure to currently available antipseudomonal therapies.

**TABLE 1:** Genotypic features and baseline MICs against β-lactams, β-lactam/β-lactamase inhibitor combinations, and comparator agents of parental strains used in this study^*a*^

				MIC (mg/L)													
Ref. ID	MLST	Infection source	β-lactamase	Ceftazidime- avibactam	Cefepime	Ceftolozane-tazobactam	Piperacillin-tazobactam	Meropenem	Aztreonam	Ceftazidime	Imipenem	Colistin	Amikacin	Gentamicin	Tobramycin	Levofloxacin	Tigecycline
1	17	BSI	OXA-50, PDC-8	2	2	0.5	4	0.25	8	2	1	0.5	4	2	0.5	0.25	8
2	845	PIHP	OXA-50, PDC-8	0.12	4	0.5	0.5	0.03	0.12	0.5	0.06	0.25	16	8	2	4	16
3	244	PIHP	OXA-847, PDC-1	1	1	0.5	2	1	2	2	1	2	4	2	0.5	0.5	8
4	244	BSI	OXA-847, PDC-1	1	1	0.5	2	0.5	2	1	1	1	2	0.5	0.25	0.25	4
5	348	PIHP	OXA-494, PDC-5	1	1	0.5	2	0.5	4	1	1	1	1	0.25	0.25	2	1
6	274	BSI	OXA-486, PDC-24	2	2	0.5	4	0.25	8	4	1	0.5	4	2	0.5	0.25	8
7	155	NA	OXA-396, PDC-5	1	1	0.5	2	0.25	4	1	1	0.5	2	1	0.25	1	8

^*a*^ Ref. ID, parental strain reference ID; MLST, multilocus sequence type; BSI, bloodstream infection; PIHP, pneumoniae in hospitalized patients; OXA, oxacillinase, intrinsic OXA-50-type; PDC, *Pseudomonas-*derived cephalosporinase, intrinsic AmpC.

## RESULTS

**Fig 1:**
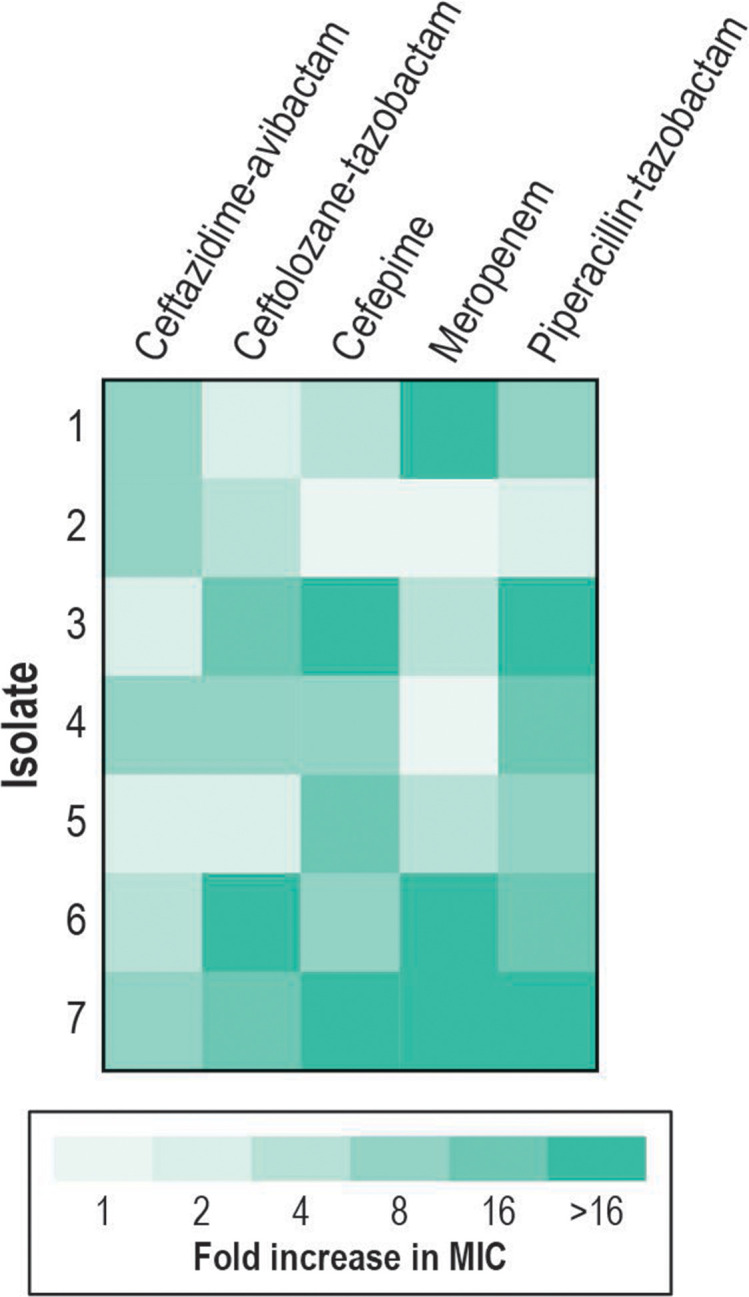
Fold change in MIC results for isolates exposed to each agent when compared to its baseline isolate.

Following 10 days of serial passaging and stepwise increases in β-lactam concentration, exposed isolates displayed MIC increases of twofold to eightfold for ceftazidime-avibactam (mean, 5.7-fold) and 2- to 32-fold for ceftolozane-tazobactam (mean, 11.4-fold; Fig. 1). The MIC increases for meropenem (1- to 128-fold; mean, 38-fold), cefepime (1- to 32-fold; mean, 14.4-fold), and piperacillin-tazobactam (2- to 256-fold; mean 52.9-fold) were slightly higher when compared to the newer β-lactam/β-LICs.

To determine whether exposure to different β-lactam or β-lactam/β-LICs affected the susceptibility to other β-lactams or agents from other antimicrobial classes (e.g., aminoglycosides, quinolones), exposed isolates were susceptibility tested against a broad set of antimicrobial agents. Overall, regardless of selective antimicrobial during lineage evolution, more exposed isolates exhibited >2-fold increase in MIC relative to their respective parent strains for ceftazidime-avibactam (24 isolates; Table 2), cefepime (n=26), piperacillin-tazobactam (n=26), and meropenem (n=26) when compared to ceftolozane-tazobactam (14 isolates). Many exposed isolates also displayed elevated MIC values (>2 fold) against ceftazidime and aztreonam (23 and 25 isolates, respectively). Still, only a few isolates developed MIC results that would be categorized as resistant or intermediate to the β-lactam agents/combinations tested except for aztreonam (17 resistant and 8 intermediate). Against non-β-lactam agents, 9 and 11 exposed isolates displayed >2- or 2-fold MIC increases for levofloxacin, respectively, including 12 isolates that presented intermediate MIC values for this agent and 6 that were resistant.

**TABLE 2:** Changes in MIC values for *P. aeruginosa* isolates exposed to β-lactam agents^*a*^

Exposed isolates MIC change	Ceftazidime- avibactam	Cefepime	Ceftolozane-tazobactam	Piperacillin-tazobactam	Meropenem	Aztreonam	Ceftazidime	Imipenem	Colistin	Amikacin	Gentamicin	Tobramycin	Levofloxacin	Tigecycline
All exposed isolates (35)														
MIC increase >2-fold	24	26	14	26	26	25	23	8	2	0	3	0	9	6
MIC increase 2-fold	6	3	14	4	5	8	6	9	4	5	4	7	11	6
Resistant MIC	5	6	1	9	7	17	7	5	2	0	0	0	6	0
Intermediate MIC	0	5	4	8	9	8	3	3	0	0	2	0	12	0
Isolates exposed to ceftazidime-avibactam (7)														
MIC increase >2-fold	5	5	1	6	6	6	4	0	0	0	1	0	4	4
MIC increase 2-fold	2	1	4	1	1	1	3	2	1	1	0	1	1	0
Resistant MIC	1	0	0	1	0	4	0	0	0	0	0	0	1	0
Intermediate MIC	0	1	0	2	3	2	1	0	0	0	1	0	4	0
Isolates exposed to cefepime (7)														
MIC increase >2-fold	4	6	4	3	5	4	5	0	0	0	1	0	2	2
MIC increase 2-fold	2	0	3	1	1	2	1	1	2	3	2	3	3	2
Resistant MIC	1	2	0	1	3	3	3	0	0	0	0	0	2	0
Intermediate MIC	0	2	1	1	1	1	1	0	0	0	1	0	1	0
Isolates exposed to ceftolozane-tazobactam (7)														
MIC increase >2-fold	6	6	5	5	6	6	6	2	0	0	1	0	1	0
MIC increase 2-fold	0	0	2	2	1	1	1	2	1	0	0	1	2	2
Resistant MIC	2	2	1	2	2	3	2	0	0	0	0	0	1	0
Intermediate MIC	0	2	2	2	1	3	0	2	0	0	0	0	2	0
Isolates exposed to meropenem (7)														
MIC increase >2-fold	2	3	1	6	3	3	2	5	2	0	0	0	1	0
MIC increase 2-fold	2	1	1	0	1	3	0	1	0	0	1	1	2	1
Resistant MIC	0	0	0	3	0	3	0	5	2	0	0	0	1	0
Intermediate MIC	0	0	0	1	1	0	0	0	0	0	0	0	3	0
Isolates exposed to piperacillin-tazobactam (7)														
MIC increase >2-fold	7	6	3	6	6	6	6	1	0	0	0	0	1	0
MIC increase 2-fold	0	1	4	0	1	1	1	3	0	1	1	1	3	1
Resistant MIC	1	2	0	2	2	4	2	0	0	0	0	0	1	0
Intermediate MIC	0	0	1	2	3	2	1	1	0	0	0	0	2	0

^*a*^ MIC, minimum inhibitory concentration.

*P. aeruginosa* isolates exposed to ceftazidime-avibactam displayed >2-fold increased MIC values for various β-lactam agents, except ceftolozane-tazobactam (1 isolate) and imipenem (none). A total of 5/7 isolates exposed to ceftazidime-avibactam had increased levofloxacin MIC results and 4/7 had a >2-fold increase in the tigecycline MIC values.

Similar to ceftazidime-avibactam, most lineages exposed to cefepime produced daughters exhibiting elevated MIC values ≥2-fold for all β-lactams except imipenem; however, in this instance, all seven isolates displayed elevated ceftolozane-tazobactam MICs (Table 2). Two-fold increases in the MIC values for amikacin, levofloxacin, tigecycline, and tobramycin were noted for 3–5 isolates exposed to cefepime.

Interestingly, most lineages evolved under ceftolozane-tazobactam selection produced terminal strains displaying ≥2-fold increases in MIC values for all other β-lactams tested. This increase was noted for 6–7 of the isolates exposed to ceftolozane-tazobactam when tested against different β-lactam agents/combinations, except for imipenem. Isolates exposed to piperacillin-tazobactam also displayed high MIC values for most other β-lactams, but to a lesser extent when compared to ceftolozane-tazobactam. Exposure to meropenem leads to the development of elevated MIC values (>2-fold) against imipenem in 5/7 isolates, and all 5 developed resistant MIC values against imipenem. Despite some increased levofloxacin MIC values, MIC increases against other classes were less common for ceftolozane-tazobactam, meropenem, and piperacillin-tazobactam when compared to ceftazidime-avibactam and cefepime.

Our experiments established a difference in the terminal susceptibility phenotypes in lineages of *P. aeruginosa* exposed to more recently developed therapeutic strategies (e.g., ceftolozane-tazobactam, ceftazidime-avibactam) versus older treatment options (e.g., meropenem, cefepime, piperacillin-tazobactam) in terms of cross-resistance to other β-lactam, β-lactam/β-lactamase inhibitor combinations, and other antimicrobial classes. We then sought to determine shared evolutionary trajectories among lineages relative to the exposure agent and performed WGS followed by an analysis of the accumulated genetic changes, specifically single nucleotide polymorphisms (SNPs) and insertions/deletions (indels). We limited our analysis to strains displaying >2-fold increases in MIC against their respective exposure agent. Five isolates exposed to ceftazidime-avibactam exhibited MICs >2-fold their parent strain, and two of these isolates possessed nonsense or missense mutations in *nalD*, a regulator of the MexAB-OprM efflux system (Table 3). In addition, one isolate displayed an alteration in the intragenic region upstream of *nalC,* which is also involved in the repression of *mexAB-oprM*. These *nalC/nalD* mutants exhibited increases in MIC values of 2-fold to 8-fold for ceftazidime-avibactam, cefepime, and ceftazidime and 4-fold to 16-fold for meropenem, piperacillin-tazobactam, and aztreonam compared to their baseline MIC. Expectedly, these isolates displayed no significant increase in imipenem MIC results as this carbapenem is not a substrate of MexAB-OprM. All these isolates displayed elevated levofloxacin (≥2-fold), two had elevated tigecycline (4-fold to 8-fold) and one had gentamicin elevated MIC values (4-fold). The remaining isolates exposed to ceftazidime-avibactam displayed elevated MIC values for most other β-lactams agents/combinations tested and displayed alterations in a gene encoding a carboxy terminal-processing peptidase, *algO,* or a putative glycosyltransferase family 4 protein-like gene.

**TABLE 3:** MIC fold change and genetic alterations in *P. aeruginosa* isolates exposed to β-lactam agents^*a*^

		Fold change in MIC compared to the parent isolate														
Parent	Selection agent	Ceftazidime-avibactam	Cefepime	Ceftolozane-tazobactam	Piperacillin-tazobactam	Meropenem	Aztreonam	Ceftazidime	Imipenem	Colistin	Amikacin	Gentamicin	Tobramycin	Levofloxacin	Tigecycline	Gene altered (amino acid change/gene name/position)
1	Ceftazidime-avibactam	8	8	4	8	8	8	8	0.5	2	1	1	1	8	4	Glycosyltransferase family 4 protein-like (Thr254Ala)
2	Ceftazidime-avibactam	8	1	2	2	2	2	2	2	1	1	1	1	1	0.5	Carboxy terminal-processing peptidase (*algO*; Ser239Pro)
4	Ceftazidime-avibactam	8	8	2	16	16	16	8	2	0.5	2	4	2	4	8	*nalD* (Tyr48Stop)
6	Ceftazidime-avibactam	4	4	2	4	8	4	2	1	1	1	1	1	4	4	*clpA* (Gly544Ser)
	Ceftazidime-avibactam															Upstream of *nalC* (+130 bp)
7	Ceftazidime-avibactam	8	8	2	16	16	8	8	1	0.25	0.25	0.12	0.5	2	0.5	*nalD* (His56Pro)
1	Cefepime	1	4	2	1	1	0.5	1	1	2	2	2	2	4	2	*amrR* (Asp155Gly)
3	Cefepime	8	32	8	128	4	16	64	1	0.5	1	1	1	1	1	*ampD* (Val10Gly)
																Glycosyltranferase (Asn214Ser)
4	Cefepime	2	8	2	8	1	2	8	1	1	2	4	2	4	4	*N*-acetyl sugar amidotransferase (*wbpG*; Val179Ala)
5	Cefepime	8	16	8	128	16	16	64	1	0.25	1	1	1	2	2	Upstream of *nalC* (+144 bp)
6	Cefepime	2	8	16	128	2	16	32	0.5	1	2	2	2	2	1	*mpl* (ΔE254-F259)
7	Cefepime	16	32	8	16	8	4	16	0.25	2	1	1	1	2	4	*cpxS* (Thr163Pro)
																*mexB* (Arg620Leu)
																*mlaE* (Ala122Glu)
																Upstream of *nalC* (+131 bp)
2	Ceftolozane-tazobactam	16	4	4	4	2	4	4	2	2	0.5	0.5	0.25	4	2	*secY* (Leu365Pro)
3	Ceftolozane-tazobactam	32	64	16	256	8	32	128	4	0.12	0.12	0.06	0.25	0.5	0.12	*mpl* (Val124Gly)
4	Ceftolozane-tazobactam	8	16	8	32	16	16	8	4	0.25	1	4	2	2	2	*galU* (Arg101Ser)
																*cpxS* (Thr163Pro)
																Hypothetical protein (His24Pro)
6	Ceftolozane-tazobactam	32	32	32	128	16	16	64	2	0.5	0.12	0.06	0.25	1	0.12	*mpl* (Ala159Thr)
7	Ceftolozane-tazobactam	8	8	16	8	16	4	8	1	0.5	0.5	0.5	1	2	1	*fstI* (Ala547Pro)
																*cpxS* (Thr163Pro)
1	Piperacillin-tazobactam	4	4	4	8	8	4	4	0.25	1	0.5	0.5	0.5	2	1	*merR-*family (PA2737; Arg36Cys)
																*pgi* (Gln306Stop)
3	Piperacillin-tazobactam	8	32	8	64	8	8	32	1	0.5	1	1	1	0.5	1	*mupP* (Tyr188Stop)
4	Piperacillin-tazobactam	8	8	2	16	16	16	8	1	0.25	0.12	0.25	0.5	4	1	*nalC* (Thr24Pro)
5	Piperacillin-tazobactam	4	4	2	8	8	4	8	4	0.25	1	1	1	1	1	*pepA* (Trp414Arg)
6	Piperacillin-tazobactam	4	4	2	16	16	4	4	2	0.25	0.12	0.06	0.25	2	0.5	*mpl* (Thr325Pro)
7	Piperacillin-tazobactam	32	64	16	256	8	16	256	2	1	2	2	2	2	2	*ampD* (Leu40Pro)
																*galU* (Tyr207His)
																Upstream of *mexR* (+184 bp)
1	Meropenem	4	4	2	4	64	4	4	8	16	1	0.5	1	1	0.5	Upstream of *nalC* (+143 bp)
																*phoP* (Thr79Ser)
3	Meropenem	1	2	1	1	4	2	1	16	0.5	1	1	1	1	1	*gacS* (Ala530Glu)
																UDP-glucose 6-dehydrogenase (PA3559; Ile224Val)
4	Meropenem	1	1	0.5	1	4	2	1	8	4	1	2	2	1	1	*oprD* (Ser40Asn/Ser278Pro)
5	Meropenem	1	1	1	2	4	1	1	1	1	1	1	1	2	1	*mexR* (+205 bp)-*mexA* (+70 bp) intergenic region
																*rpoB* (ΔE282-Q289)
6	Meropenem	2	4	1	4	128	4	1	16	1	0.5	1	1	8	2	*ftsI* (Val471Gly)
																*nalC* (Gly132Glu)
7	Meropenem	8	8	4	16	64	8	8	8	0.5	0.25	0.125	0.5	2	0.5	*nalC* (Arg48Gly)

^*a*^ MIC, minimum inhibitory concentration.

Of the six cefepime-exposed isolates selected for sequencing, one isolate displayed an alteration in the TetR family transcriptional regulator *amrR* (Table 3). This isolate only displayed a 4-fold increase in the cefepime and levofloxacin MIC values. Another isolate had a mutation in *ampD* along with a missense alteration in a glycosyltransferase gene. This isolate exhibited elevated MIC values for all β-lactams ranging from 8-fold to 128-fold and no increase in MIC results for other classes. One isolate displaying elevated MIC values for levofloxacin, gentamicin, tigecycline, ceftazidime, and piperacillin-tazobactam in addition to cefepime had an alteration in *wbpG,* an *N*-acetyl sugar amidotransferase involved in LPS biosynthesis (20, 21). An 18 bp deletion in *mpl* was detected in one isolate which was resistant to ceftazidime, piperacillin- and ceftolozane-tazobactam but not carbapenems or ceftazidime-avibactam. The *mpl* gene encodes a murein peptide ligase and is involved in the recycling of the cell wall peptidoglycan (22). The remaining isolate harbored alterations in *mexB*, upstream of *nalC, mlaE,* and *cpxS*. Alterations in CpxS have been observed in isolates exposed to different antimicrobial agents alone or in combination (23). This isolate exhibited increased (≥2-fold) MIC values for levofloxacin and tigecycline and most β-lactams but not imipenem.

Of five ceftolozane-tazobactam-exposed isolates sequenced, two strains acquired identical alterations in CpxS (Thr163Pro; Table 3). One of these isolates also possessed a missense mutation in *ftsI,* encoding penicillin-binding protein (PBP) 3, while the other isolate carried mutations in *galU*, involved in LPS biosynthesis (24), and a hypothetical protein. The three other isolates exposed to ceftolozane-tazobactam carried mutations in the secretion protein *secY* (one isolate; Leu365Pro) or *mpl* (two isolates; Val124Gly or Ala159Thr). Three of the five sequenced isolates exposed to ceftolozane-tazobactam were also noted for increased susceptibility to ≥2 aminoglcyoside agents and/or colistin; these isolates possessed alterations in *mpl* (n=2) and *secY, ftsI,* and *cpxS*.

Multiple isolates exposed to piperacillin-tazobactam acquired mutations in genes and intergenic regions comparable to those identified under different selection regimes (Table 3). Unique alterations among these isolates were identified in a putative MerR-family transcriptional regulator homologous to PA2737 from *P. aeruginosa* PAO1, the glucose-6-phosphate isomerase Pgi, the protease PepA, and the peptidoglycan recycling-associated MupP. Levofloxacin MICs were elevated ≥2-fold in most of these isolates. Similar to those terminal isolates produced under ceftolozane-tazobactam selection, piperacillin-tazobactam-selected isolates displayed increased susceptibility to colistin (4/6 isolates) and aminoglycosides (3/6 isolates; Table 3).

Out of the six meropenem mutants sequenced, one displayed alterations in OprD (Ser40Asn/Ser278Pro) and increased 8-fold and 4-fold increases in MIC values for imipenem and colistin, respectively (Table 3). Three isolates contained alterations within or upstream of *nalC* observed along with alterations in the PBP3 (Val471Gly) or the transcriptional regulator PhoP (Thr79Ser). The isolate harboring alterations in *phoP* and the upstream region of *nalC* had elevated MIC values for all β-lactams, except ceftolozane-tazobactam and colistin (16-fold increase). The two isolates with *nalC* missense mutations displayed twofold or eightfold increases in levofloxacin MIC values. One isolate displayed an alteration in the intergenic region between *mexR* and *mexA* along with a mutation in *rpoB*. The last isolate displayed alterations in the two-component system sensor histidine kinase GacS and a putative UDP-glucose 6-dehydrogenase homologous to PA3559 from *P. aeruginosa* PAO1.

## DISCUSSION

*P. aeruginosa* was the fourth most common cause of bloodstream infections among isolates submitted during the initial 20 years of the SENTRY Antimicrobial Surveillance Program, contributing 5.3% of the isolates analyzed (25). This organism is an important cause of nosocomial pneumonia infections, and its prevalence is higher among immunocompromised and burn patients. *P. aeruginosa* isolates are intrinsically resistant to many antimicrobial genes and the accumulation of mutations modulating the activities of intrinsic resistance mechanisms is more frequently identified among *P. aeruginosa* clinical isolates compared to other species (1).

In this study, we exposed *P. aeruginosa* clinical isolates from bloodstream or pneumonia infections displaying low baseline MIC values to common antipseudomonal agents to a 10-day serial passaging with one of five β-lactam agents, including new β-lactam/β-LICs, to identify mechanisms of resistance selected during exposure to these agents. We also evaluated the changes in susceptibility to various other antimicrobial agents of the same (β-lactam) and diverse classes (e.g., aminoglycosides, colistin). Overall, the mean MIC values displayed greater increases for meropenem, cefepime, and piperacillin-tazobactam when compared to ceftazidime-avibactam and ceftolozane-tazobactam. We also noted reduced susceptibility against β-lactams other than the agent of exposure as well as agents from other antimicrobial classes (Table 2). Generally, reduced susceptibility to non-passaging antibiotics occurred equally for all agents, although evolution under meropenem selection produced fewer terminal strains with reduced susceptibility to other agents. It is noteworthy that the increased MIC values displayed by terminal isolates generally did not rise to resistant levels, except for aztreonam (17/35 isolates; 48.6%). Furthermore, isolates passaged under selection with piperacillin-tazobactam or ceftolozane-tazobactam generated daughter strains with increased susceptibility to colistin and/or multiple aminoglycosides. We observed no pattern in the accumulation of mutations based on isolate, sequence type, or infection source (data not shown).

The most common genetic alterations detected in the terminal isolates were those in or upstream (+131, 133, 143, or 144 bp) of *nalC* and *nalD,* which are involved in the regulatory pathway of the *mexAB-oprM* efflux system (Table 3); alterations in the *mexR-mexA* intergenic region were noted in two isolates, as was a single isolate with substitutions in AmrR or MexB. Increased levofloxacin and unchanged impenem MIC results were noted in most isolates displaying *nalC*, *nalD,* or *mexR* alterations. Overexpression of *mexAB-oprM* confers resistance to all β-lactams, except for imipenem, and the quinolones (26). Furthermore, these MexAB-OprM regulatory alterations were noted in isolates exposed to all agents but ceftolozane-tazobactam and isolates harboring only alterations in these targets usually displayed <2-fold increase in the ceftolozane-tazobactam MIC values. In studies evaluating the transcription of different genes in clinical isolates resistant to β-lactams, *mexAB-oprM* overexpression did not seem to affect the susceptibility of ceftolozane-tazobactam (27), corroborating our results.

Despite not being affected by alterations affecting *mexAB-oprM* expression, isolates exposed to ceftolozane-tazobactam developed elevated MIC values for other β-lactam agents *via* mechanisms common to the lineages exposed to other β-lactam or β-lactam/β-LIC pressure. Mutations leading to amino acid substitutions and deletions in Mpl, identified in four isolates exposed to ceftolozane-tazobactam (*n* = 2) and cefepime or piperacillin-tazobactam (one isolate each), were the second most common alteration observed. Mutations in *mpl* have been frequently cited in *in vitro* studies as a common mutational site resulting in overexpression *ampC* (28) . Mutations in *mpl* were also associated with increased susceptibility to colistin and aminoglycosides (Table 3).

Overexpression of the PDC-encoding gene has been reported as a cause of resistance to newer β-lactam/β-LICs in clinical isolates (8, 15) and isolates exposed *in vivo* to these agents (29); multiple isolates possessed alterations in AmpD, mutations in which increase PDC expression (30), following exposure to piperacillin-tazobactam and cefepime (one isolate each), but not for other agents. Interestingly, the piperacillin-tazobactam-exposed isolate displaying a single amino acid substitution in AmpD also exhibited alterations in GalU and the upstream region of *mexR* and it is possible the additive effect of these alterations underlies the development of resistance in this isolate (8-fold to 256-fold increases in MIC results for different β-lactams).

Finally, numerous isolates obtained during our passaging experiments against all selection agents acquired mutations in genes related to cell wall and outer membrane homeostasis. Multiple alterations were noted in genes encoding PBP3, OprD, PhoP, ClpA, Pgi, and CpxS, all of which are known to contribute to β-lactam resistance in *P. aeruginosa* isolates (1, 31). In addition, multiple genes of unknown or presumed function were identified in our study, some of which were the only alterations present in the terminal isolate and may influence the reduction of susceptibility in those strains by as-yet undiscovered mechanisms. Future work will focus on defining the role of those proteins and if their role in the development of resistance is shared among other clinical *P. aeruginosa* isolates.

Overall, our results demonstrate following exposure to meropenem, cefepime, and piperacillin-tazobactam terminal isolates displayed greater fold increases in MIC values compared to the isolates obtained after exposure to ceftolozane-tazobactam and ceftazidime-avibactam. This data may indicate these newer agents could help prevent the emergence of high-level resistance.

## MATERIALS AND METHODS

### Isolates, antimicrobial exposure, and susceptibility testing

Seven *P. aeruginosa* isolates including six clinical isolates from patients suffering bloodstream infection (*n* = 3) or pneumonia (*n* = 3) were selected as parental strains. The susceptibility reference strain ATCC 27853 was also included as a parental strain. Baseline MIC values were determined in triplicate by the reference broth microdilution method (32). All parental strains were exposed to ceftazidime-avibactam, ceftolozane-tazobactam, meropenem, cefepime, and piperacillin-tazobactam. All β-lactamase inhibitors were tested at a 4 mg/L fixed concentration. A summary of the susceptibility profiles and associated genotypic information for all parental strains is shown in Table 1. Strains were generally maintained on tryptic soy agar with 5% sheep’s blood (BAP, Remel; Lenexa, KS) and susceptibility testing was performed using cation-adjusted Mueller-Hinton broth. For serial passaging, lineages for each drug-parent combination were initiated in an identical fashion as was performed for the determination of baseline MICs against each agent; however, lineages were only grown in singlicate. Following overnight incubation, the entire contents of the well with the highest concentration of each antimicrobial displaying visible growth were used to prepare a new 0.5 McFarland standard. This inoculum was diluted to a final concentration of 5 × 10^5^ CFU/mL in a new panel with the same antimicrobial agent. This process was repeated for nine additional days. A single colony was selected and passaged twice on drug-free BAP prior to further testing and storage.

Terminal isolates were susceptibility tested by reference broth microdilution method (32) against all agents used in exposure and comparator agents.

### Whole-genome sequencing

Terminal isolates with >2-fold changes from the baseline and their respective parental strains were sequenced using short-read sequencing methods; parental strains were also sequenced using long-read technology. To obtain short-read sequencing data, total genomic DNA (1 ng) was used as input for DNA library construction using the Nextera XT library construction protocol and index kits (Illumina; San Diego, California, USA) following the manufacturer’s instructions. Libraries were sequenced on a MiSeq Sequencer (Illumina) using the MiSeq Reagent Kit v3 (600cycle).

High molecular weight DNA was obtained using the Nanobind CBB Big DNA Kit (PacBio, Menlo Park, CA, USA) according to the manufacturer’s instructions. Purified genomic DNA was allowed to solubilize at room temperature for 16 h. Purity was assessed using a NanoDrop 2000 spectrophotometer (Thermo Fisher Scientific) and DNA concentration was measured using the Invitrogen Qubit 1X dsDNA HS Assay Kit (Thermo Fisher Scientific) on a Life Technologies Qubit 3.0 Fluorometer (Thermo Fisher Scientific).

Sequencing library preparation was carried out with Rapid Barcoding Sequencing Kit (SQK-RBK0004) using ~50 ng input DNA per sample on an R9.4.1 (FLO-MIN106) flow cell using the MinION Mk1C sequencer controlled by MinKNOW version 5.3.6 (Oxford Nanopore Technologies Ltd, Oxford, UK). Library preparation and sequencing were performed according to the manufacturer’s protocol. Sequencing reactions were run for 48 h; base calling was performed with Guppy version 6.4.2.

### Bioinformatic analysis

For parental strains, hybrid assemblies were created by assembling short- and long-read Unicycler v0.4.8-beta (33), which builds an initial assembly graph from short reads using the *de novo* assembler SPAdes (34) and then simplifies the graph using information from short and long reads. Variant calling between the parent-daughter pairs only utilized short-read sequencing from the daughter isolates; variant calling was performed using MAUVE V2.4.0 (35). All SNPs determined by MAUVE were confirmed by mapping quality trimmed reads independently to the baseline assembly. Reads were mapped using BWA v0.7.12-r1039 (36). Insertion/deletion (indel) sites were realigned using IndelRealigner from the GATK toolbox v3.8 (37). High confidence variants were called by samtools v1.8 and filtered by bcftools v1.8 (38). Filtering criteria for the variant call format (VCF) file were as follows: a minimum read depth of 4 (≥2 reads per strand), >30 map quality, >50 average base quality, no significant strand bias, and >75% of mutations within reads to support the presence of any given alteration. Repeat regions of >50 bp were removed from VCF using MUMmer v3.0 (39). Baseline assembly was annotated using Prokka v1.14.0 (40). SNPs were annotated using Snpeff 4.3t (41). Short reads were subjected to quality trimming using a sliding window threshold of Q18. The reference sequence was built using the Unicycler’s default parameters and all parental assemblies produced fully circularized, single contigs (data not shown). SNP quality metrics then were applied (34). All potential SNPs were confirmed using reference-guided alignments using DNASTAR (DNASTAR, Inc; Madison, WI). Quality metrics for individual variant calls on a per-isolate basis are displayed in Table S1.

## SUPPLEMENTAL MATERIAL

Supplemental Table S1 (Quality metrics for reported variants identified in this study) aac.01363-23-s0001.docx Quality metrics for NGS.

ASM does not own the copyrights to Supplemental Material that may be linked to, or accessed through, an article. The authors have granted ASM a non-exclusive, world-wide license to publish the Supplemental Material files. Please contact the corresponding author directly for reuse.

## References

[1] Lister PD, Wolter DJ, Hanson ND. 2009. Antibacterial-resistant Pseudomonas aeruginosa: clinical impact and complex regulation of chromosomally encoded resistance mechanisms. Clin Microbiol Rev 22:582–610. doi:10.1128/CMR.00040-09 19822890 PMC2772362

[2] Magiorakos AP, Srinivasan A, Carey RB, Carmeli Y, Falagas ME, Giske CG, Harbarth S, Hindler JF, Kahlmeter G, Olsson-Liljequist B, Paterson DL, Rice LB, Stelling J, Struelens MJ, Vatopoulos A, Weber JT, Monnet DL. 2012. Multidrug-resistant, extensively drug-resistant and pandrug-resistant bacteria: an international expert proposal for interim standard definitions for acquired resistance. Clin Microbiol Infect 18:268–281. doi:10.1111/j.1469-0691.2011.03570.x 21793988

[3] Shortridge D, Gales AC, Streit JM, Huband MD, Tsakris A, Jones RN. 2019. Geographic and temporal patterns of antimicrobial resistance in Pseudomonas aeruginosa over 20 years from the SENTRY antimicrobial surveillance program, 1997-2016. Open Forum Infect Dis 6:S63–S68. doi:10.1093/ofid/ofy343 30895216 PMC6419917

[4] Prevention CDC. 2019. Pseudomonas aeruginosa in healthcare settings. Available from: https://www.cdc.gov/hai/organisms/pseudomonas.html

[5] Bassetti M, Vena A, Sepulcri C, Giacobbe DR, Peghin M. 2020. Treatment of bloodstream infections due to Gram-negative bacteria with difficult-to-treat resistance. Antibiotics (Basel) 9:632. doi:10.3390/antibiotics9090632 32971809 PMC7558339

[6] Blomquist KC, Nix DE. 2021. A critical evaluation of newer β-lactam antibiotics for treatment of Pseudomonas aeruginosa infections. Ann Pharmacother 55:1010–1024. doi:10.1177/1060028020974003 33228374

[7] Yahav D, Giske CG, Grāmatniece A, Abodakpi H, Tam VH, Leibovici L. 2020. New β-lactam-β-lactamase inhibitor combinations. Clin Microbiol Rev 34:e00115-20. doi:10.1128/CMR.00115-20 PMC7667665 33177185

[8] Slater CL, Winogrodzki J, Fraile-Ribot PA, Oliver A, Khajehpour M, Mark BL. 2020. Adding insult to injury: mechanistic basis for how AmpC mutations allow Pseudomonas aeruginosa to accelerate cephalosporin hydrolysis and evade avibactam. Antimicrob Agents Chemother 64:e00894-20. doi:10.1128/AAC.00894-20 32660987 PMC7449160

[9] Ruedas-López A, Alonso-García I, Lasarte-Monterrubio C, Guijarro-Sánchez P, Gato E, Vázquez-Ucha JC, Vallejo JA, Fraile-Ribot PA, Fernández-Pérez B, Velasco D, Gutiérrez-Urbón JM, Oviaño M, Beceiro A, González-Bello C, Oliver A, Arca-Suárez J, Bou G. 2022. Selection of AmpC beta-lactamase variants and metallo-beta-lactamases leading to ceftolozane/tazobactam and ceftazidime/avibactam resistance during treatment of MDR/XDR Pseudomonas aeruginosa infections. Antimicrob Agents Chemother 66:e0206721. doi:10.1128/AAC.02067-21 34930034 PMC8846482

[10] Arca-Suárez J, Vázquez-Ucha JC, Fraile-Ribot PA, Lence E, Cabot G, Martínez-Guitián M, Lasarte-Monterrubio C, Rodríguez-Iglesias M, Beceiro A, González-Bello C, Galán-Sánchez F, Oliver A, Bou G. 2020. Molecular and biochemical insights into the in vivo evolution of AmpC-mediated resistance to ceftolozane/tazobactam during treatment of an MDR Pseudomonas aeruginosa infection. J Antimicrob Chemother 75:3209–3217. doi:10.1093/jac/dkaa291 32728723

[11] Barnes MD, Taracila MA, Rutter JD, Bethel CR, Galdadas I, Hujer AM, Caselli E, Prati F, Dekker JP, Papp-Wallace KM, Haider S, Bonomo RA. 2018. Deciphering the evolution of cephalosporin resistance to ceftolozane-tazobactam in Pseudomonas aeruginosa. mBio 9:e02085-18. doi:10.1128/mBio.02085-18 30538183 PMC6299481

[12] Winkler ML, Papp-Wallace KM, Hujer AM, Domitrovic TN, Hujer KM, Hurless KN, Tuohy M, Hall G, Bonomo RA. 2015. Unexpected challenges in treating multidrug-resistant Gram-negative bacteria: resistance to ceftazidime-avibactam in archived isolates of Pseudomonas aeruginosa. Antimicrob Agents Chemother 59:1020–1029. doi:10.1128/AAC.04238-14 25451057 PMC4335889

[13] Castanheira M, Mills JC, Farrell DJ, Jones RN. 2014. Mutation-driven beta-lactam resistance mechanisms among contemporary ceftazidime-nonsusceptible Pseudomonas aeruginosa isolates from U.S. hospitals. Antimicrob Agents Chemother 58:6844–6850. doi:10.1128/AAC.03681-14 25182652 PMC4249397

[14] Castanheira M, Johnson MG, Yu B, Huntington JA, Carmelitano P, Bruno C, Rhee EG, Motyl M. 2021. Molecular characterization of baseline Enterobacterales and Pseudomonas aeruginosa isolates from a phase 3 nosocomial pneumonia (ASPECT-NP) clinical trial. Antimicrob Agents Chemother 65:e02461-20. doi:10.1128/AAC.02461-20 33318005 PMC8092525

[15] Castanheira M, Doyle TB, Smith CJ, Mendes RE, Sader HS. 2019. Combination of MexAB-OprM overexpression and mutations in efflux regulators, PBPs and chaperone proteins is responsible for ceftazidime/avibactam resistance in Pseudomonas aeruginosa clinical isolates from US hospitals. J Antimicrob Chemother 74:2588–2595. doi:10.1093/jac/dkz243 31225882

[16] Gomis-Font MA, Pitart C, Del Barrio-Tofiño E, Zboromyrska Y, Cortes-Lara S, Mulet X, Marco F, Vila J, López-Causapé C, Oliver A. 2021. Emergence of resistance to novel cephalosporin-beta-lactamase inhibitor combinations through the modification of the Pseudomonas aeruginosa MexCD-OprJ efflux pump. Antimicrob Agents Chemother 65:e0008921. doi:10.1128/AAC.00089-21 34060900 PMC8284450

[17] Arca-Suárez J, Lasarte-Monterrubio C, Rodiño-Janeiro B-K, Cabot G, Vázquez-Ucha JC, Rodríguez-Iglesias M, Galán-Sánchez F, Beceiro A, González-Bello C, Oliver A, Bou G. 2021. Molecular mechanisms driving the in vivo development of OXA-10-mediated resistance to ceftolozane/tazobactam and ceftazidime/avibactam during treatment of XDR Pseudomonas aeruginosa infections. J Antimicrob Chemother 76:91–100. doi:10.1093/jac/dkaa396 33083833

[18] Fraile-Ribot PA, Del Rosario-Quintana C, López-Causapé C, Gomis-Font MA, Ojeda-Vargas M, Oliver A. 2019. Emergence of resistance to novel beta-lactam-beta-lactamase inhibitor combinations due to horizontally acquired AmpC (FOX-4) in Pseudomonas aeruginosa sequence type 308. Antimicrob Agents Chemother 64:e02112-19. doi:10.1128/AAC.02112-19 31685471 PMC7187601

[19] Fraile-Ribot PA, Mulet X, Cabot G, Del Barrio-Tofiño E, Juan C, Pérez JL, Oliver A. 2017. in vivo emergence of resistance to novel cephalosporin-β-Lactamase inhibitor combinations through the duplication of amino acid D149 from OXA-2 β-Lactamase (OXA-539) in sequence type 235 Pseudomonas aeruginosa. Antimicrob Agents Chemother 61:e01117-17. doi:10.1128/AAC.01117-17 28674059 PMC5571340

[20] Burrows LL, Charter DF, Lam JS. 1996. Molecular characterization of the Pseudomonas aeruginosa serotype O5 (PAO1) B-band lipopolysaccharide gene cluster. Mol Microbiol 22:481–495. doi:10.1046/j.1365-2958.1996.1351503.x 8939432

[21] Choi DS, Kim DK, Choi SJ, Lee J, Choi JP, Rho S, Park SH, Kim YK, Hwang D, Gho YS. 2011. Proteomic analysis of outer membrane vesicles derived from Pseudomonas aeruginosa. Proteomics 11:3424–3429. doi:10.1002/pmic.201000212 21751344

[22] Das D, Hervé M, Feuerhelm J, Farr CL, Chiu H-J, Elsliger M-A, Knuth MW, Klock HE, Miller MD, Godzik A, Lesley SA, Deacon AM, Mengin-Lecreulx D, Wilson IA. 2011. Structure and function of the first full-length murein peptide ligase (Mpl) cell wall recycling protein. PLoS One 6:e17624. doi:10.1371/journal.pone.0017624 21445265 PMC3060825

[23] Barbosa C, Mahrt N, Bunk J, Graßer M, Rosenstiel P, Jansen G, Schulenburg H. 2021. The genomic basis of rapid adaptation to antibiotic combination therapy in Pseudomonas aeruginosa. Mol Biol Evol 38:449–464. doi:10.1093/molbev/msaa233 32931584 PMC7826179

[24] Imperi F, Ciccosanti F, Perdomo AB, Tiburzi F, Mancone C, Alonzi T, Ascenzi P, Piacentini M, Visca P, Fimia GM. 2009. Analysis of the periplasmic proteome of Pseudomonas aeruginosa, a metabolically versatile opportunistic pathogen. Proteomics 9:1901–1915. doi:10.1002/pmic.200800618 19333994

[25] Diekema DJ, Hsueh PR, Mendes RE, Pfaller MA, Rolston KV, Sader HS, Jones RN. 2019. The microbiology of bloodstream infection: 20-year trends from the SENTRY antimicrobial surveillance program. Antimicrob Agents Chemother 63:e00355-19. doi:10.1128/AAC.00355-19 31010862 PMC6591610

[26] Masuda N, Sakagawa E, Ohya S, Gotoh N, Tsujimoto H, Nishino T. 2000. Substrate specificities of MexAB-OprM, MexCD-OprJ, and MexXY-oprM efflux pumps in Pseudomonas aeruginosa. Antimicrob Agents Chemother 44:3322–3327. doi:10.1128/AAC.44.12.3322-3327.2000 11083635 PMC90200

[27] Castanheira M, Doyle TB, Hubler CM, Collingsworth TD, DeVries S, Mendes RE. 2022. The plethora of resistance mechanisms in Pseudomonas aeruginosa: transcriptome analysis reveals a potential role of lipopolysaccharide pathway proteins to novel β-lactam/β-lactamase inhibitor combinations. J Glob Antimicrob Resist 31:72–79. doi:10.1016/j.jgar.2022.07.021 35931381

[28] Fournier D, Carrière R, Bour M, Grisot E, Triponney P, Muller C, Lemoine J, Jeannot K, Plésiat P, GERPA Study Group. 2021. Mechanisms of resistance to ceftolozane/tazobactam in Pseudomonas aeruginosa: results of the GERPA multicenter study. Antimicrob Agents Chemother 65:e01117-20. doi:10.1128/AAC.01117-20 PMC7849014 33199392

[29] Lahiri SD, Johnstone MR, Ross PL, McLaughlin RE, Olivier NB, Alm RA. 2014. Avibactam and class C β-lactamases: mechanism of inhibition, conservation of the binding pocket, and implications for resistance. Antimicrob Agents Chemother 58:5704–5713. doi:10.1128/AAC.03057-14 25022578 PMC4187909

[30] Juan C, Moyá B, Pérez JL, Oliver A. 2006. Stepwise upregulation of the Pseudomonas aeruginosa chromosomal cephalosporinase conferring high-level beta-lactam resistance involves three AmpD homologues. Antimicrob Agents Chemother 50:1780–1787. doi:10.1128/AAC.50.5.1780-1787.2006 16641450 PMC1472203

[31] Sanz-García F, Hernando-Amado S, Martínez JL. 2018. Mutation-driven evolution of Pseudomonas aeruginosa in the presence of either ceftazidime or ceftazidime-avibactam. Antimicrob Agents Chemother 62:e01379-18. doi:10.1128/AAC.01379-18 30082283 PMC6153820

[32] CLSI. 2018. Methods for dilution antimicrobial susceptibility tests for bacteria that grow aerobically. In M07, 11th ed. Clinical and Laboratory Standards Institute, Wayne, PA.

[33] Wick RR, Judd LM, Gorrie CL, Holt KE. 2017. Unicycler: Resolving bacterial genome assemblies from short and long sequencing reads. PLoS computational biology 13:e1005595. doi:10.1371/journal.pcbi.1005595 28594827 PMC5481147

[34] Bankevich A, Nurk S, Antipov D, Gurevich AA, Dvorkin M, Kulikov AS, Lesin VM, Nikolenko SI, Pham S, Prjibelski AD, Pyshkin AV, Sirotkin AV, Vyahhi N, Tesler G, Alekseyev MA, Pevzner PA. 2012. SPAdes: a new genome assembly algorithm and its applications to single-cell sequencing. J Comput Biol 19:455–477. doi:10.1089/cmb.2012.0021 22506599 PMC3342519

[35] Darling ACE, Mau B, Blattner FR, Perna NT. 2004. Mauve: Multiple alignment of conserved Genomic sequence with Rearrangements. Genome research 14:1394–1403. doi:10.1101/gr.2289704 15231754 PMC442156

[36] Li H, Durbin R. 2009. Fast and accurate short read alignment with burrows-Wheeler transform. Bioinformatics (Oxford, England) 25:1754–1760. doi:10.1093/bioinformatics/btp324 19451168 PMC2705234

[37] McKenna A, Hanna M, Banks E, Sivachenko A, Cibulskis K, Kernytsky A, Garimella K, Altshuler D, Gabriel S, Daly M, DePristo MA. 2010. The genome analysis Toolkit: A Mapreduce framework for analyzing next-generation DNA sequencing data. Genome research 20:1297–1303. doi:10.1101/gr.107524.110 20644199 PMC2928508

[38] Li H. 2011. A statistical framework for SNP calling, Mutation discovery, Association mapping and population Genetical parameter estimation from sequencing data. Bioinformatics (Oxford, England) 27:2987–2993. doi:10.1093/bioinformatics/btr509 21903627 PMC3198575

[39] Kurtz S, Phillippy A, Delcher AL, Smoot M, Shumway M, Antonescu C, Salzberg SL. 2004. Versatile and open software for comparing large Genomes. Genome biology 5:R12. doi:10.1186/gb-2004-5-2-r12 14759262 PMC395750

[40] Seemann T. 2014. Prokka: Rapid Prokaryotic genome annotation. Bioinformatics (Oxford, England) 30:2068–2069. doi:10.1093/bioinformatics/btu153 24642063

[41] Cingolani P, Platts A, Wang LL, Coon M, Nguyen T, Wang L, Land SJ, Lu X, Ruden DM. 2012. A program for Annotating and predicting the effects of single nucleotide Polymorphisms, Snpeff: SNPs in the genome of Drosophila Melanogaster strain W1118; Iso-2; Iso-3. Fly 6:80–92. doi:10.4161/fly.19695 22728672 PMC3679285

