# Supplementary material for: In vitro development of resistance against antipseudomonal agents: comparison of novel β-lactam/β-lactamase inhibitor combinations and other β-lactam agents
Source: Antimicrob Agents Chemother. 2024 Mar 25;68(5):e01363-23. doi: 10.1128/aac.01363-23 (PMC11064483; doi:10.1128/aac.01363-23)
Supplement: Supplemental Table S1 (Quality metrics for reported variants identified in this study) — Quality metrics for NGS. [file aac.01363-23-s0001.docx]

**Supplemental Table 1.** Quality metrics for reported variants identified in this study

| Parent | Selection Agent | Location | Supporting bases (Bases Q≥30; %)^a^ |
| --- | --- | --- | --- |
| 1 | Ceftazidime-avibactam | Putative glycosyltransferase(A-2151648-G) | 37(22; 59.5) |
| 2 | Ceftazidime-avibactam | *algO*(T-4897441-C) | 34(22; 64.7) |
| 4 | Ceftazidime-avibactam | *nalD*(G-1502588-C) | 62(46; 74.2) |
| 6 | Ceftazidime-avibactam | *nalC* upstream(A-1378186-G) | 50(34; 68.0) |
|  |  | *clpA*(G-2558856-A) | 39(25; 64.1) |
| 7 | Ceftazidime-avibactam | nalD(T-1476944-G) | 49(34; 69.4) |
| 1 | Cefepime | amrR(T-3725937-C) | 64(47; 73.4) |
| 3 | Cefepime | ampD(A-5491746-C) | 39(27; 69.2) |
|  |  | Putative glycosyltransferase(T-6058815-C) | 58(39; 67.2) |
| 4 | Cefepime | wbpG(T-1977032-C) | 28(18;64.2) |
| 5 | Cefepime | nalC upstream(A-1378715-G) | 34(20; 58.8) |
| 6 | Cefepime | mpl(CCGAGGACGGCTCGCGCTTCGAGG- 999611-CCGAGG) | 26(19; 73.1) |
| 7 | Cefepime | cpxS(G-484717-T) | 75(53; 70.7) |
|  |  | mexB(A-1274576-G) | 24(16; 66.7) |
|  |  | mlaE(T-1872345-G) | 26(14; 53.8) |
|  |  | nalC upstream(G-5410366-T)^b^ | 49(31; 63.3) |
| 2 | Ceftolozane-tazobactam | secY(A-5989576-G) | 59(38; 64.4) |
| 3 | Ceftolozane-tazobactam | mpl(T-1092741-G) | 54(35; 64.8) |
| 4 | Ceftolozane-tazobactam | cpxS(T-1915142-G) | 21(14; 66.7) |
|  |  | galU(G-3526894-T) | 48(32; 66.7) |
|  |  | Hypothetical (A-6179349-C) | 41(30; 73.1) |
| 6 | Ceftolozane-tazobactam | mpl(G-999328-A) | 33(21; 63.6) |
| 7 | Ceftolozane-tazobactam | cpxS (T-1872345-G) | 20(16; 80.0) |
|  |  | ftsI(C-5375355-G) | 51(33; 64.7) |
| 1 | Piperacillin-tazobactam | merR-family regulator PA2737(C-2585209-T) | 57(37; 64.9) |
|  |  | pgi(C-5965764-T) | 44(30; 68.2) |
| 3 | Piperacillin-tazobactam | mupP(G-2073469-T) | 55(38; 69.1) |
| 4 | Piperacillin-tazobactam | nalC (T-1342652-G) | 26(15; 57.7) |
| 5 | Piperacillin-tazobactam | pepA(A-1254924-T) | 36(24; 66.7) |
| 6 | Piperacillin-tazobactam | mpl(A-999826-C) | 44(33; 75.0) |
| 7 | Piperacillin-tazobactam | mexR upstream(C-481600-T) | 41(24; 58.5) |
|  |  | galU(A-3522191-G) | 21(13; 61.9) |
|  |  | ampD(A-5487991-G) | 24(16; 66.7) |
| 1 | Meropenem | nalC upstream(A-1490304-G) | 36(24; 66.7) |
|  |  | phoP(T-4643053-A) | 49(30; 61.2) |
| 3 | Meropenem | PA3559(T-1646498-C) | 37(25; 67.6) |
|  |  | gacS(C-4940057-A) | 35(24; 68.6) |
| 4 | Meropenem | oprD(G-4684707-A) | 60(33; 55.0) |
|  |  | oprD(T-4685420-C) | 52(29; 55.8) |
| 5 | Meropenem | mexR upstream(G-475369-A) | 79 (71;89.9) |
|  |  | rpoB (ACCAGCTGGAAAAGGCTGGCGTGAGCCAGCTGGAA -751861-ACCAGCTGGAA) | 37(32; 86.5) |
| 6 | Meropenem | nalC(C-1377662-T) | 33(21; 63.6) |
|  |  | ftsI(A-4989991-C) | 24(16; 66.7) |
| 7 | Meropenem | nalC(G-1274305-C) | 59(36; 61.0) |

^a^Depth of coverage of bases supporting the variant call, including the number of bases with a quality (Q) score greater than or equal to 30 (%).

^b^ phred-scaled QUAL score, 84. All other reported variant calls produced QUAL scores of 225.
